# Deep metagenomic characterization of the gut virome in pregnant women with preeclampsia

**DOI:** 10.1128/msphere.00676-23

**Published:** 2024-03-20

**Authors:** Li-Juan Lv, Ji-Ying Wen, Yue Zhang, Ruo-Chun Guo, Hui Li, Zhou-Ting Yi, Tian-Wen He, Min-Chai Chen, Yang Chen, Xiao-Yan Wu, Sheng-hui Li, Jian Kang, Ya-Ping Hou, Qiu-long Yan, Ai-Hua Yin

**Affiliations:** 1 Medical Genetic Center, Guangdong Women and Children Hospital, Guangzhou, China; 2 Department of Obstetrics, Guangdong Women and Children Hospital, Guangzhou, China; 3 Puensum Genetech Institute, Wuhan, China; 4 Department of Microbiology, College of Basic Medical Science, Dalian Medical University, Dalian, China; University of Michigan-Ann Arbor, Ann Arbor, Michigan, USA

**Keywords:** preeclampsia, gut virome, gut microbiota, viral diversity, viral function, pregnant women

## Abstract

Preeclampsia (PE), a pregnancy-specific syndrome, has been associated with the gut bacteriome. Here, to investigate the impact of the gut virome on the development of PE, we identified over 8,000 nonredundant viruses from the fecal metagenomes of 40 early-onset PE and 37 healthy pregnant women and profiled their abundances. Comparison and correlation analysis showed that PE-enriched viruses frequently connected to *Blautia* species enriched in PE. By contrast, bacteria linked to PE-depleted viruses were often the Bacteroidaceae members such as *Bacteroides* spp., *Phocaeicola* spp., *Parabacteroides* spp., and *Alistipes shahii*. In terms of viral function, PE-depleted viruses had auxiliary metabolic genes that participated in the metabolism of simple and complex polysaccharides, sulfur metabolism, lipopolysaccharide biosynthesis, and peptidoglycan biosynthesis, while PE-enriched viruses had a gene encoding cyclic pyranopterin monophosphate synthase, which seemed to be special, that participates in the biosynthesis of the molybdenum cofactor. Furthermore, the classification model based on gut viral signatures was developed to discriminate PE patients from healthy controls and showed an area under the receiver operating characteristic curve of 0.922 that was better than that of the bacterium-based model. This study opens up new avenues for further research, providing valuable insights into the PE gut virome and offering potential directions for future mechanistic and therapeutic investigations, with the ultimate goal of improving the diagnosis and management of PE.

**IMPORTANCE:**

The importance of this study lies in its exploration of the previously overlooked but potentially critical role of the gut virome in preeclampsia (PE). While the association between PE and the gut bacteriome has been recognized, this research takes a pioneering step into understanding how the gut virome, represented by over 8,000 nonredundant viruses, contributes to this condition. The findings reveal intriguing connections between PE-enriched viruses and specific gut bacteria, such as the prevalence of *Blautia* species in individuals with PE, contrasting with bacteria linked to PE-depleted viruses, including members of the Bacteroidaceae family. These viral interactions and associations provide a deeper understanding of the complex dynamics at play in PE.

## INTRODUCTION

Preeclampsia (PE) is a pregnancy-specific syndrome typically characterized by hypertension and proteinuria. Its global prevalence among pregnant populations ranges from 2% to 8% (1). PE, if not managed properly, can progress to eclampsia, with these conditions collectively contributing to 10% to 15% of maternal mortality (2). The pathogenesis of PE is multifaceted and is linked to various risk factors such as chronic hypertension, diabetes mellitus, pre-pregnancy body mass index (BMI), antiphospholipid syndrome, and a family history of the condition (3–5). Moreover, an increasing body of evidence underscored a significant association between gut microbiota and PE. In a prior study conducted in 2019, we revealed gut bacterial dysbiosis in pregnant women with early-onset PE during the third trimester. This dysbiosis was characterized by elevated levels of *Blautia*, *Bilophila*, *Fusobacterium*, and *Ruminococcus2*, alongside reductions in *Akkermansia*, *Dialister*, *Faecalibacterium*, *Gemmiger*, and *Methanobrevibacter* (6). This dysbacteriosis persisted postpartum at 1 and 6 weeks. Another study confirmed that gut microbiota of PE patients could induce PE-like symptoms in mice via fecal microbiota transplantation (7). Notably, the bacterial composition in the gut of PE pregnant women, as identified by 16S rRNA sequencing, echoed our findings (7). In our most recent investigation, we conducted deep shotgun metagenomic sequencing to provide further insights into the functional attributions of prokaryotes associated with early-onset PE (8). Our results suggested that certain bacteria may contribute to the stability of the gut microbiota in PE and underscored the potential of bacterial markers as clinical targets for early-onset PE, offering new perspectives on both the pathogenesis and clinical involvement of PE.

Some epidemiological and case-control studies have suggested associations between PE and viral populations, including adeno-associated virus-2 (9), cytomegalovirus (10), Epstein–Barr virus (11), human herpes virus (12), herpes simplex virus (13), and human immunodeficiency virus infections (14). A plausible explanation for these links is that viral infections stimulate the production of pro-inflammatory cytokines (e.g., IFN-γ, IL-12, and TNF-ɑ) and oxidative stress. These factors can lead to impaired extravillous cytotrophoblast invasion and endothelial dysfunction in individuals with PE (15, 16). Furthermore, gut viral populations constitute significant components of the intestinal microbial ecology and are intimately intertwined with human health. The gut viral homeostasis is carefully maintained under normal conditions; however, it can be disrupted when individuals experience health issues such as inflammatory bowel disease (17), colorectal cancer (18), and immune disorders (19–21). Notably, a study has even connected the gut virome of women of childbearing age to polycystic ovary syndrome (22). Strikingly, the gut virome of pregnant women with PE has remained largely unexplored thus far. Considering the altered gut bacteriome in PE patients and the intricate interplay between the gut virome and bacteriome, exploring the gut virome of PE patients becomes imperative.

Herein, we embarked on the characterization of the gut virome in PE by reanalyzing public deep shotgun metagenomic sequencing data (8) from 40 pregnant women with early-onset PE and 37 healthy pregnant women. By this thorough reanalysis, we successfully recovered over 8,000 nonredundant viruses from the metagenomic samples and conducted a comprehensive profiling of the gut virome based on these nonredundant viruses. Notably, our investigation revealed a substantial difference in the composition of the gut viral community between PE patients and their healthy counterparts. A total of 158 nonredundant viruses exhibited significant variations in relative abundance between these two groups. Furthermore, our findings underscore a close relationship between these gut viral populations and gut bacteria, and notably, we identified that gut viral signatures possess superior potential for distinguishing the PE state from healthy individuals compared to the bacteriome.

## RESULTS

### Construction of the nonredundant virus catalog from fecal metagenomes

**Fig 1:**
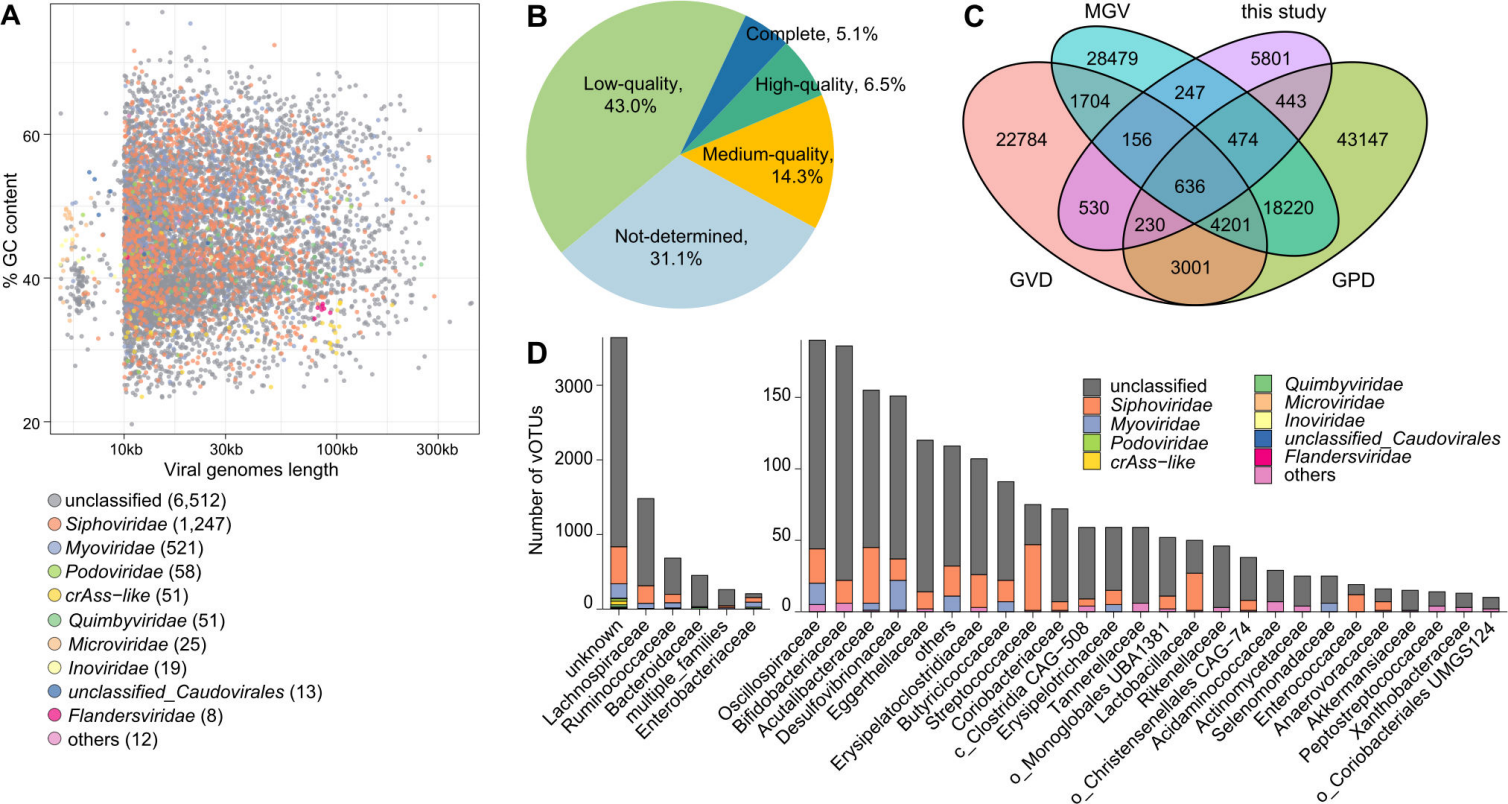
Overview of the nonredundant gut virus catalog. (**A**) Scatter plot showing the distribution of vOTUs by genome length and % GC content. (**B**) Pie plot showing the distribution of the estimated quality of the vOTUs. (**C**) Venn plot showing the overlap of the current virus catalog and the other three published gut virus catalogs. (**D**) Distribution of taxonomic assignments and prokaryotic hosts of the gut virus catalog. The hosts of vOTUs are grouped at the family level, and the family-level viral taxa are labeled by colors.

To explore the gut virome of PE, we analyzed the deep shotgun metagenomic sequencing data set of 40 pregnant women with early-onset PE and 37 healthy pregnant women from our previous study , which represented a total of 781.0 Gb of high-quality non-human data (on average, 10.1 Gb per sample). Metagenomic assembly of the fecal samples generated a total of 446,117 contigs with a minimum length threshold of 5,000 bp (total length 8.3 Gbp, N50 length 31,417 bp; Table S1). A comprehensive analysis workflow is outlined in Fig. S1. Using the previously developed methodologies based on both homology features and machine learning methods (see Materials and Methods) (23, 24), we recognized 33,902 highly credible viral sequences from the contigs. Subsequently, we organized these viruses into species-level clusters, employing criteria of >95% nucleotide similarity and >75% pairwise overlap. This analysis yielded a nonredundant catalog of 8,517 viral operational taxonomic units (vOTUs). The lengths of these nonredundant vOTUs ranged from 5,008 bp to 431,572 bp, with an average length of 31,340 bp and N50 length of 45,100 bp (Fig. 1A; Table S2). Based on the completeness evaluation by CheckV (25), 5.1% and 6.5% of the vOTUs were complete viruses or viruses with high-quality genomes (completeness >90%), respectively, and 14.3% of them were viruses with medium-quality genomes with completeness of 50%–90% (Fig. 1B), whereas the remaining vOTUs were mainly viruses with low (completeness <50%) (43.0%) or undetermined quality (31.1%). Remarkably, only 31.9% (2,716/8,517) of the vOTUs in our catalog had been identified in combination with several currently published human gut virome databases, including Gut Phage Database (26), Gut Virome Database (27), and Metagenomic Gut Virus catalog (28) (Fig. 1C), which highlighted the exceptional novelty of our gut virus catalog.

Taxonomically, a majority (76.5%) of the nonredundant vOTUs could not be assigned to a known family. A total of 14.6% and 6.1% of the vOTUs were classified into *Siphoviridae* and *Myoviridae,* respectively (Fig. 1A; Table S2), which are two of the most prevalent families within the human gut virome (29). The remaining viruses were allocated to less common viral families such as *Podoviridae*, crAss-like, *Quimbyviridae*, *Microviridae*, *Inoviridae*, and *Flandersviridae*. A total of 4,878 vOTUs, equivalent to 57.3% of the nonredundant virus catalog, were linked to one or multiple prokaryotic hosts using established methodologies (see Materials and Methods). Predominantly, bacterial taxa such as Lachnospiraceae, Ruminococcaceae, Bacteroidaceae, and Enterobacteriaceae emerged as the most frequent hosts of these gut viruses (Fig. 1D; Table S2).

### Structural variations of the gut virome in PE patients

**Fig 2:**
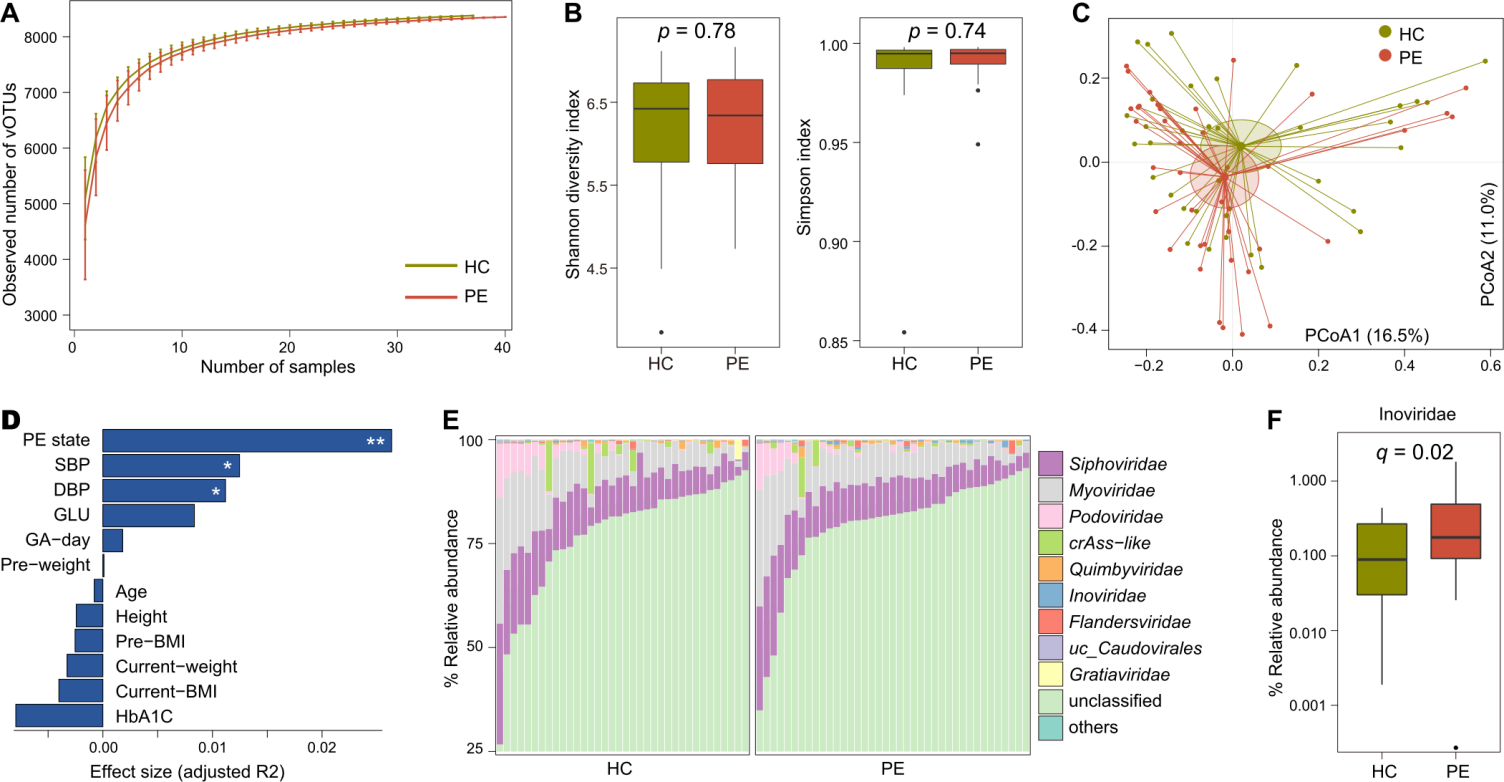
Diversity and compositional analyses of the gut virome in PE patients and healthy controls. (**A**) Rarefaction analysis showing an increase in the number of vOTUs observed as the number of random samples increased. (**B**) Boxplot showing the Shannon diversity index (left panel) and Simpson index (right panel) of patients and controls. (**C**) Principal coordinate analysis (PCoA) of the Bray–Curtis distance of the gut virome of all samples at the vOTU level. Samples are shown at the first and second principal coordinates (PCoA1 and PCoA2), and the ratio of variance contributed by these two principal coordinates is shown. Lines connect samples in the same group, and circles cover samples near the center of gravity for each group. (**D**) Permutational multivariate analysis of variance (PERMANOVA) showing the effect size of phenotypic and clinical parameters that contribute to the variance of the overall gut virome. Bar plots indicate the explained variation (adjusted R2) of each factor. Adonis test with 1,000 permutations: *, *P* < 0.05; **, *P* < 0.01. (**E**) Bar plot showing the gut viral composition of fecal metagenomes from PE patients and healthy controls at the family level. Only the top 10 viral families with the highest abundance are shown. (**F**) Boxplot showing the relative abundance of differentially abundant viral families between the two groups. For panels **B** and **F**, boxes represent the interquartile range between the first and third quartiles and the median (internal line). Whiskers denote the lowest and highest values within 1.5 times the range of the first and third quartiles, respectively; dots represent outlier samples beyond the whiskers. PE, preeclampsia patients; HC, healthy controls; SBP, systolic blood pressure; DBP, diastolic blood pressure; GLU, fasting blood glucose; GA-day, gestational age (day); Pre-BMI, pre-pregnancy body mass index.

We performed a rarefaction curve analysis to show the variation of gut viral richness estimated by the observed number of vOTUs. This analysis revealed that there were no significant differences in viral richness between PE patients and healthy controls at the same sample sizes (Fig. 2A). Also, both the Shannon diversity index and Simpson index exhibited no substantial distinctions between the two groups (Student’s *t*-test, *P* > 0.05; Fig. 2B; Table S3).

PCoA based on the Bray–Curtis distances at the vOTU level revealed that the gut virome of PE patients and healthy controls was visibly separated (Fig. 2C). This result was further corroborated by the PERMANOVA, which disclosed that the PE state explained 2.6% of the overall structure variations of the gut virome among all samples, with adonis *P* = 0.009. As a comparison, the endogenetic and clinical parameters of the subjects, such as age, BMI, and fasting glucose, exhibited relatively minor impacts on the gut virome (with effect sizes < 1% and adonis *P* > 0.05 for these parameters; Fig. 2D). Additionally, two PE-related clinical parameters, systolic blood pressure and diastolic blood pressure, had a limited yet statistically significant influence on the gut virome. These results highlighted a considerable alteration in the gut virome of PE patients when compared with that of healthy controls.

At the family level, we found that the known viral families accounted for only 21.7% of the total abundance of the gut virome in all samples. Among these, *Siphoviridae* (average relative abundance 9.6 ± 5.4% in all samples) and *Myoviridae* (8.5 ± 6.0%) were the main components, followed by *Podoviridae* (1.6 ± 2.8%), crAss-like (0.9 ± 2.3%), *Quimbyviridae* (0.3 ± 0.5%), and *Inoviridae* (0.3 ± 0.3%; Fig. 2E; Table S4). Of these families, only *Inoviridae* showed a significant difference with an increasing trend in the virome of PE patients compared with healthy controls (Mann–Whitney U test *q* = 0.02; Fig. 2F).

### Identification of PE-associated viral signatures and their functions

**Fig 3:**
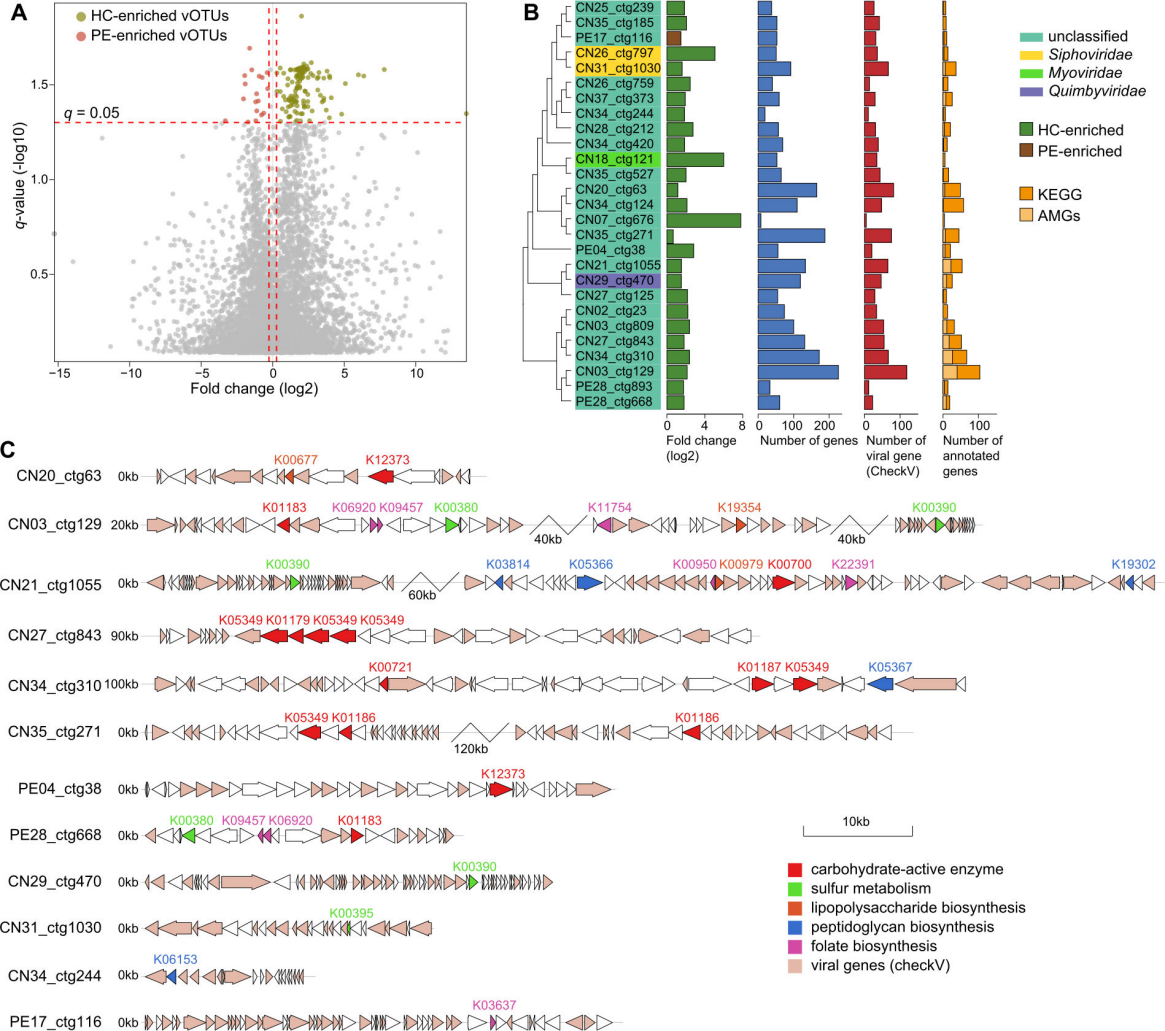
PE-associated gut viral signatures and functions. (**A**) Volcano map showing the fold change and *q*-values of all vOTUs. vOTUs with absolute value of fold change greater than 1.2 and *q*-value less than 0.05 were considered to be significantly different between PE patients and healthy controls, represented by red and green dots in the figure, respectively. (**B**) Detailed information of 27 PE-associated vOTUs with medium-quality, high-quality, or complete genomes. For each vOTU, the numbers of total genes, viral genes (estimated by CheckV), and Kyoto Encyclopedia of Genes and Genomes (KEGG)-annotated genes are shown. (**C**) Genome structure of the PE-associated vOTUs contained genes involving polysaccharide metabolism, sulfur metabolism, lipopolysaccharide and peptidoglycan biosynthesis, and folate biosynthesis. PE, preeclampsia patients; HC, healthy controls.

Using the Mann–Whitney U test with false discovery rate (FDR) correction (*q* < 0.05), we identified 158 vOTUs that significantly differed in relative abundances between PE patients and healthy controls (Fig. 3A; Table S5). Twenty of these vOTUs were enriched in the virome of PE patients, and 138 of them were enriched in controls. The PE-enriched vOTUs included 1 *Siphoviridae* virus, 1 *Myoviridae* virus, and 18 family-level unclassified viruses, while the control-enriched vOTUs included 6 *Siphoviridae*, 6 *Myoviridae*, and 2 *Quimbyviridae* viruses and 124 unclassified viruses.

To describe the functional characteristics of the PE-associated viruses, we next especially focused on the PE-associated vOTUs exhibiting medium-quality, high-quality, or complete genomes, including 1 PE-enriched vOTU (medium-quality) and 26 control-enriched vOTUs (2 complete, 8 high-quality, and 16 medium-quality viruses) (Fig. 3B). We predicted the protein-coding genes of these vOTUs and annotated their functions based on the Kyoto Encyclopedia of Genes and Genomes (KEGG) database. Out of the 2,280 genes with these vOTUs, 32.7% could be assigned to KEGG orthologs (KOs), with most of these genes reflecting typical viral functions involving integrase, transposase, DNA replication and repair, and prokaryotic defense systems (Table S6). Strikingly, among the annotated genes, 189 were recognized as viral auxiliary metabolic gene (AMGs) with potential metabolic capacity. An exploration of these AMGs led to several interesting findings. First, we found that eight control-enriched vOTUs encoded several carbohydrate-active enzymes (Fig. 3C; Table S6). These carbohydrate-active enzymes included beta-glucosidase (K05349), hexosaminidase (K12373), chitinase (K01183), sialidase (K01186), and endoglucanase (K01179), and they were enabled to decompose the simple and complex polysaccharides such as galactose, glucan, starch, chitin, and sphingolipids in the human gut. Second, five control-enriched vOTUs harbored genes that participated in sulfur metabolism, especially the phosphoadenosine phosphosulfate reductase (K00390), adenylylsulfate reductase (K00395), and sulfite reductase (K00380) that involve sulfate/sulfite reduction (Fig. 3C; Table S6). Third, we found that several enzymes involved in the biosynthesis of lipopolysaccharide (LPS) or peptidoglycan also frequently appeared, spanning in five vOTUs that were enriched in the virome of healthy subjects. Of particular note, the PE-enriched vOTU (PE17_ctg116) possessed a distinctive gene, cyclic pyranopterin monophosphate synthase (K03637), which participates in the biosynthesis of the molybdenum cofactor.

### Correlations between gut viral and bacterial signatures

**Fig 4:**
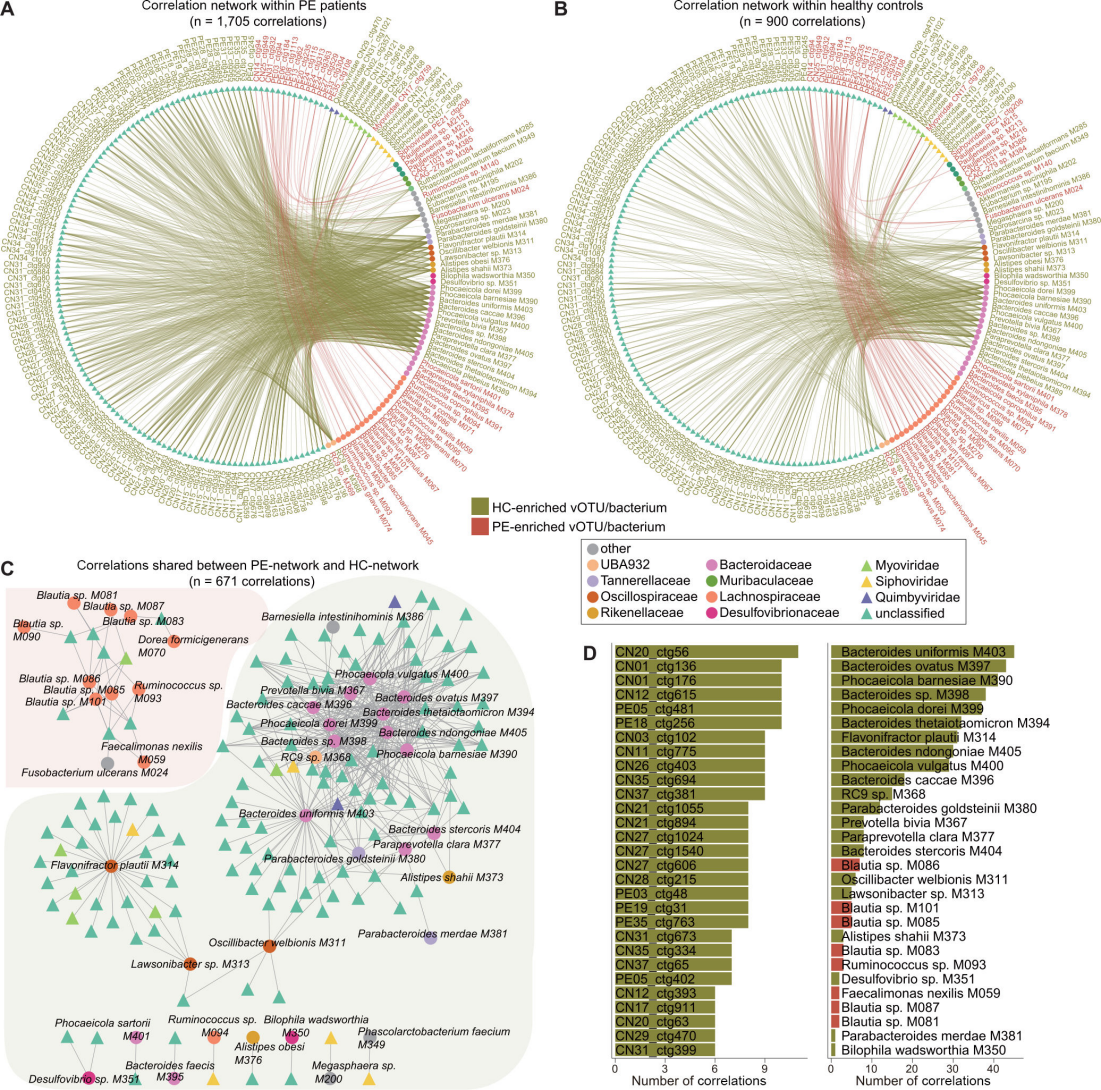
Correlation analysis between PE-associated viruses and bacterial species. (**A and B**) Network showing the virus–bacterium correlations in PE patients (**A**) and healthy controls (**B**). (**C**) Sharing correlations between PE and control networks. (**D**) Bar charts showing the top 20 viruses (left panel) and bacteria (right panel) with the largest number of correlations in the sharing network. For panels **A**, **B**, and **C**, viruses and bacteria are colored based on their family-level taxonomic assignment. For panels **A**, **B**, and **D**, the virus and bacteria names are colored by their enrichment in PE patients (red) and healthy controls (green). PE, preeclampsia patients; HC, healthy controls.

In our previous study focused on the bacteriome, 74 bacterial species were identified as associated with PE (30). To delve deeper into the intricate relationship between viruses and bacteria, we carried out a Spearman correlation analysis between 158 PE-associated vOTUs and 74 PE-associated bacteria. Two virus–bacterium correlation networks containing 1,705 and 900 correlations were generated in the PE patients and healthy controls, respectively (Spearman’s correlation coefficient >0.6; Fig. 4A and B; Table S7). This separation was done to minimize the potential influence of the disease state on the correlations. Remarkably, a substantial majority of the correlations in the control network (74.6%, 671 out of 900) were also present in the PE network (Fig. 4C), suggesting that many virus–bacteria associations can still be maintained in disease states. However, there were numerous virus–bacterium correlations that specifically occurred in the PE patients. An example was *Barnesiella intestinihominis* M386, which had connected to 58 vOTUs in the PE patients but only one vOTU in controls (Table S7), suggesting that this bacterium was more likely to interact with the viruses in patients. These findings further supported the idea that the PE condition not only influences the composition of the virome and bacteriome but also their interactions.

We next especially noted the 671 shared correlations between the PE and control networks, which involved 138 vOTUs and 44 bacterial species. A high proportion of these correlations occurred between the PE-depleted Bacteroidaceae members (including *Bacteroides* spp., *Phocaeicola* spp., *Parabacteroides* spp., and *Alistipes shahii*) and some PE-depleted unclassified vOTUs (Fig. 4C and D). Also, the PE-depleted species *Flavonifractor plautii* M314 had connected to many PE-depleted vOTUs including four *Myoviridae* viruses. Conversely, some PE-enriched *Blautia* species had frequently connected to several vOTUs. These findings suggested the potential central roles of these bacterial taxa in terms of interacting with PE-associated viruses.

### Diagnostic potential of the gut viral and bacterial signatures

**Fig 5:**
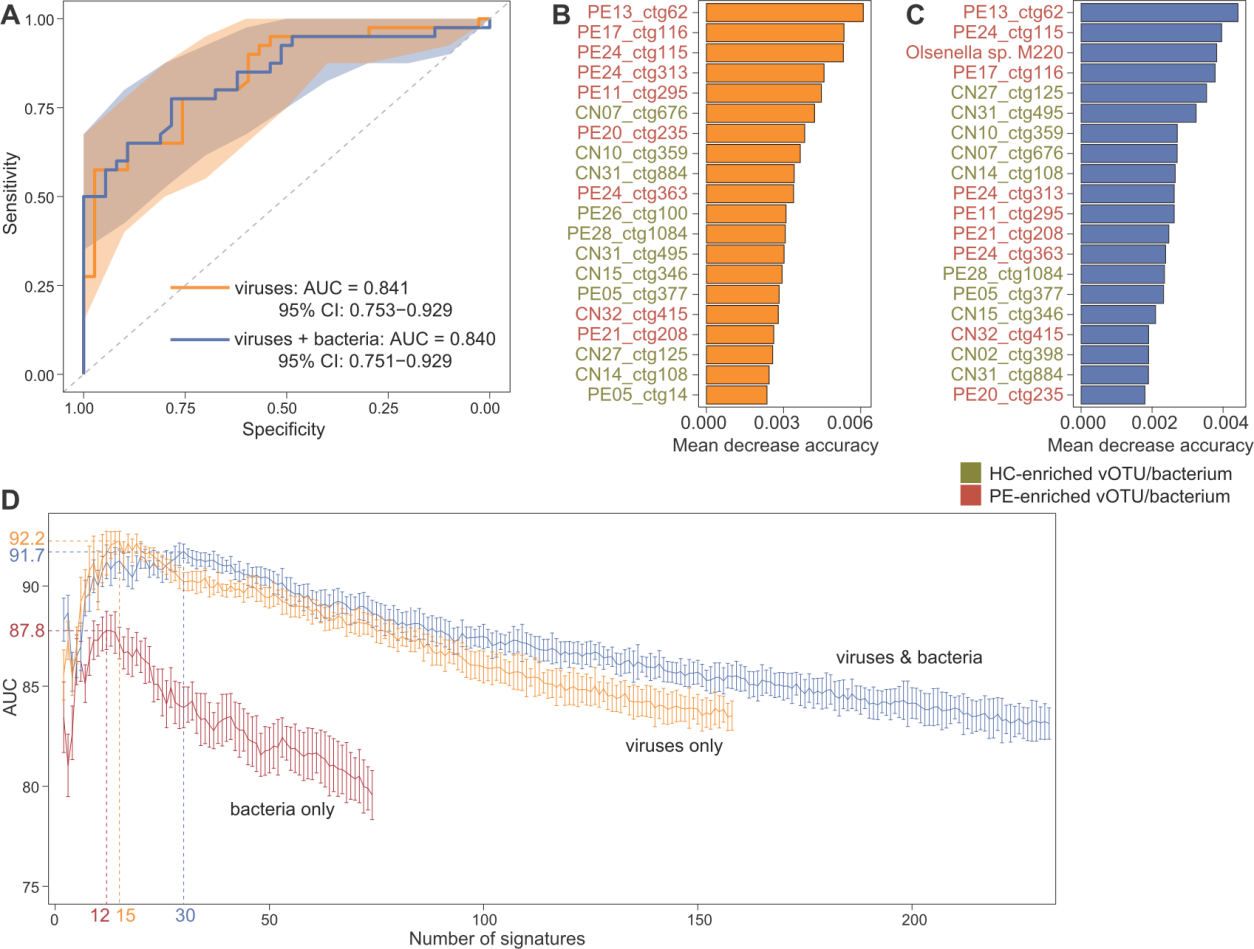
Classification of patients and controls based on gut viral and bacterial signatures. (**A**) Random forest models for discriminating PE patients and healthy controls based on the gut viral signatures (viral model) and both viral and bacterial signatures (virus–bacterium combined model). The AUC and 95% CI are shown. (**B and C**) Mean decrease in the accuracy of the 20 most important signatures in the viral model (**B**) and virus–bacterium combined model (**C**). Viruses and bacteria that are enriched in patients and controls are labeled with red and green colors, respectively. (**D**) Exploring the classification performance for different numbers of microbial signatures ordered in importance. Nodes show the average AUC of models with 10 repetitions under a specified number of vOTUs, and the error bars show the square deviations. PE, preeclampsia patients; HC, healthy controls.

Finally, in order to evaluate the capacity of the gut virome in distinguishing PE patients and healthy controls, we constructed a random forest model based on the relative abundances of 158 PE-associated vOTUs. This model obtained a cross-validation area under the receiver operating characteristic curve (AUC) of 0.841 (95% confidence interval [CI] 0.753–0.929; Fig. 5A) in identifying PE patients from healthy controls. Notably, this discriminatory power surpassed that of a model trained with the 74 PE-associated gut bacteria, as provided in our previous study (30). Moreover, the introduction of the gut bacterial signatures into a new model did not improve the classification ability of patients and controls (AUC = 0.840). Several PE-enriched vOTUs such as PE13_ctg62, PE17_ctg116, and PE24_ctg115 had the highest importance scores in the viral model, while a PE-enriched species *Olsenella* sp. M220 was among the most important signatures in the virus–bacterium combined model (Fig. 5B and C; Table S8). In addition, we also evaluated models based on individual endogenetic and clinical parameters for their ability to differentiate between PE and healthy cases. However, we found that models utilizing these parameters did not yield significant improvements when compared to the viral composition-based model (Fig. S2).

To generate a minimal set of gut microbial signatures for PE classification, we trained new random forest models using the most important viruses and bacteria. This analysis revealed that the virus model achieved the optimal AUC of 0.922 when using a subset of the top 15 important vOTUs, while the virus–bacterium combined model obtained the highest AUC of 0.917 at the number of 30 viruses/bacteria (Fig. 5D; Table S8). In comparison, a model using only bacterial signatures generated the highest AUC of 0.878 when featuring 15 bacterial species. Collectively, these findings suggested that these gut viral signatures offer the potential for identifying the state of PE.

## DISCUSSION

Preeclampsia, a complex disorder with unknown exact pathogenesis, has been frequently associated with the gut microbial community. In this study, we illustrated the differences in gut viral diversity, composition, and function between pregnant women with early-onset PE and healthy pregnant women, showing the dysbiosis of the gut viral community in PE patients. The integration analysis of the gut virome and bacteriome suggested that the development of PE was associated with interactions with viruses and bacteria.

Although there was no significant difference in gut viral richness and evenness, we observed a considerable alteration in the overall viral community structure of PE patients compared with that of healthy controls via PERMANOVA. The effect size of this alteration was larger than that of individual parameters such as age, BMI, and gestational days. For the family-level comparison, *Inoviridae* was significantly increased in PE patients. *Inoviridae* is currently understood primarily through filamentous bacteriophages that can impact biofilm formation and virulence of host bacteria and promote bacterial adaptation to the environment (31, 32). In this study, Lachnospiraceae, previously recognized as the PE-enriched bacterial family (30), is one of the main known hosts of *Inoviridae.* The members of *Inoviridae* may contribute to the development of PE by improving the growth of certain PE-enriched bacteria.

The vOTU-level comparison identified 158 PE-associated viruses. Interestingly, these viruses harbored various auxiliary metabolic genes, especially viral signatures reduced in pregnant women with early-onset PE. For example, PE-reduced (i.e., control-enriched) vOTUs could encode several carbohydrate-active enzymes involving degradation of the plant- and animal-derived saccharides. An environmental virome study has reported that viruses contain various glycoside hydrolase genes contributing to plant-derived polysaccharide degradation (33). In this study, several PE-reduced vOTUs could express sialidase and hexosaminidase, which are necessary for metabolizing human milk oligosaccharides (34). Given that maternal gut microbes could transfer to the infant microbiome (35), these PE-reduced vOTUs may promote the assembly of their host bacterium in infants. Besides, the genes of three enzymes participating in sulfate/sulfite reduction were detected in PE-reduced vOTUs. Although the mechanism of gut viral sulfur metabolism in human health remains unknown, previous studies have reported that gut bacteria frequently encode the genes involved in sulfur metabolism, which links to a variety of diseases such as gestational diabetes mellitus (36), Crohn’s disease (37), and colorectal cancer (38), which may suggest that PE-reduced vOTUs affect maternal health by participating in the sulfur metabolism of gut bacteria. We also found that PE-reduced vOTUs harbored several enzymes involved in the biosynthesis of LPS or peptidoglycans that can induce inflammatory responses in the immune system, though LPS- and peptidoglycan-encoding genes appear to be more frequently expressed by disease-enriched microbes (39–41). Taken together, our findings indicated that the PE-reduced vOTUs had broadly participated in many meaningful functions involving polysaccharide metabolism, sulfur metabolism, and synthesis of LPS and peptidoglycan, which may be directly related to the metabolic or biological processes in the human gut ecosystem, especially in healthy mothers and their infants. In addition, this study indicated that PE-enriched vOTU (PE17_ctg116), the gene cyclic pyranopterin monophosphate synthase (K03637), seemed to participate in the biosynthesis of the molybdenum cofactor. In our previous study, we revealed that the molybdenum metabolism is one of the PE-enriched KOs. The molybdate transport system is critical to bacterial virulence (42). The molybdate transport system is mainly encoded by PE-enriched *Blautia* and *Collinsella* species. However, we did not find any practically significant virus related to them.

This study still has some limitations. First, due to the high variability of the gut virome, it can be influenced by numerous host factors and external environmental factors, such as ethnicity, geographical location, diet, and lifestyle. To mitigate this, future investigations will require large sample sizes to elucidate the impact of PE on the gut virome definitively. Second, although our study provides insights into the overall alterations in the gut virome of PE patients and identifies potential PE-associated viruses, the limitations in experimental design and the complexity of PE mean that a single cross-sectional study cannot provide causality evidence. For this purpose, future longitudinal studies or direct experimental evidence will be necessary. Lastly, despite the current research confirming the distinguishability of PE-associated viruses in PE patients and healthy individuals, the unclear mechanisms of the gut virome and the challenges associated with obtaining its composition imply that further extensive research is needed for its clinical applications, such as early prediction of PE.

### Conclusion

Our finding suggested that the gut virome of pregnant women was associated with early-onset PE. Taxonomic and functional comparisons showed that the PE-associated viruses, such as *Siphoviridae, Myoviridae*, and *Quimbyviridae* viruses, may contribute to the development of PE by interacting with the host bacteriome. The classification model based on gut viral signatures provides a new perspective for the clinical diagnosis of PE patients in the foreseeable future.

## MATERIALS AND METHODS

### Pre-processing of sequencing reads

All metagenomic samples in this study were collected and provided by Lv et al. (30). Briefly, our previous study enrolled 40 pregnant women with PE and 37 healthy pregnant women. Patients with multiple pregnancies and pregnancy complications (such as fetal malformation, gestational diabetes, intrahepatic cholestasis syndrome, or chorioamnionitis) were excluded, and pregnant women with clinical conditions (such as diabetes, severe hypertension, malignancies, and infectious diseases) were also not included. Healthy pregnant women were matched to the PE group based on age, gestational age, and parity. The diagnosis of PE was conducted following the guidelines of the American College of Obstetricians and Gynecologists as outlined by Croke et al. (43). Clinical and biochemical parameters for all participants were measured following the methods described in a previous study (6). Fecal samples from both patients and healthy pregnant women were collected in late pregnancy, using sterile fecal collection containers, and promptly stored at −80°C. Total DNA from fecal samples was extracted using the TianGen Biotech fecal DNA extraction kit, following the provided instructions. DNA concentration and purity were assessed using NanoDrop 2000 and Qubit 4.0 instruments, respectively. Fecal DNA was fragmented into segments using the Covaris M220 instrument, and metagenomic sequencing libraries were constructed with the TruSeq DNA sample preparation kit. Sequencing was performed on the Illumina NovaSeq platform using paired-end 150-bp shotgun metagenomic sequencing. We first removed low-quality reads in each metagenome via fastp v0.20.1 with the parameters “-l 90 -q 20 u 30 -y --trim_poly_g” (44) and then removed human reads by mapping reads to the GRCh38 reference genome using bowtie2 v2.4.1 with the default parameters (45). The remaining reads were recognized as clean reads and used for the following analysis.

### Assembly and processing of viral sequences

The clean reads of each sample were assembled into contigs using Megahit v1.2.9 with the parameters “--k-list 21,41,61,81,101,121,141” (46). Only contigs of ≥5 kb in length were used to identify viral sequences in each sample. The identification, decontamination, and dereplication of viral sequences were performed according to the prior studies (20, 47–49). Briefly, the assembled contigs underwent a stringent quality assessment as follows: (i) contigs were classified as viral if their viral gene content surpassed that of microbial genes, as determined by CheckV v0.7.0 (25); (ii) contigs exhibiting a *P* value of less than 0.01 and a score exceeding 0.90 in DeepVirFinder v1.0 (50); and (iii) identification as viral sequences by VIBRANT v1.2.1 (51) using default settings. To ensure the purity of the viral sequences, a decontamination step, as per the approach outlined in a prior study (27), was implemented based on searching for bacterial universal single-copy orthologs (BUSCO) within each viral sequence. The ratio of BUSCO genes to the total gene count within each viral sequence (BUSCO ratio) was then calculated, and sequences with a BUSCO ratio of larger than 5% were eliminated. All viral sequences were compared in pairs through BLASTn v2.9.0 (52), and viruses exhibiting a shared nucleotide identity of 95% or greater across at least 75% of their respective sequences were clustered together to form a vOTU. The quality of viral sequences was estimated using CheckV v0.7.0 (25). Taxonomic annotation of viral sequences was executed through protein sequence alignment against a comprehensive database, which combined resources from the Virus-Host DB (53) and viral proteins from crAss-like (54), *Flandersviridae*, *Quimbyviridae*, and *Gratiaviridae* (55). A viral sequence was assigned a family-level classification when over a quarter of its proteins displayed match to the same viral family. Virus–host predictions were carried out employing two bioinformatic methodologies: CRISPR-spacer matches and prophage blasts, following the previous methodologies (23). Functional annotation of viral proteins was performed based on the KEGG database (56). Each protein received a KO assignment based on the best-hit gene in the database.

### Taxonomic profiles

To generate taxonomic profiles of gut viral communities, clean reads in each sample were mapped into reference sequences of all vOTUs using bowtie2 v2.4.1 with the parameters “--end-to-end --fast --no-unal.” Total mapping reads of all samples were randomly subsampled to the same sequencing amount (4 million). The relative abundance of each vOTU was calculated as the number of reads mapped to this vOTU divided by the total mapping reads. In addition, the relative abundance of each viral family was generated by adding up the relative abundances of vOTUs annotated with this family. The gut bacterial taxonomic profile was provided by Lv et al. (57).

### Statistical analysis

Statistical analysis and visualization were implemented in R v4.0.3 (58).

#### Alpha and beta diversity

Gut virome diversities were estimated based on the profiles at the vOTU level. The observed number of vOTUs was calculated as the number of vOTUs greater than 0 in the relative abundance. The Shannon and Simpson diversity indexes were calculated by the diversity function from the vegan (v2.6-4) package (59). The Bray–Curtis distances between samples were calculated by the vegdist function (vegan v2.6-4). PCoA of Bray–Curtis distances was implemented via the pcoa function (vegan v2.6-4). PERMANOVA was implemented using the adonis function (vegan v2.6-4).

#### Identification of viral markers

The identification of viral markers at the vOTU-level was performed between pregnant women with early-onset PE and healthy pregnant women using the wilcox.test function from the R stats package. The obtained *P* values were adjusted via the p.adjust function (stats package) with the option “method = BH.” A vOTU with an adjusted *P* value (FDR) of <0.05 was recognized as a PE-associated viral marker.

#### Correlation analysis

The correlation coefficient of gut viral and bacterial markers was measured by the cor.test function with the option “method = Spearman.” The data visualization was carried out via the R ggraph (v2.1.0) package and the Cytoscape software (v3.9.1) (60).

#### Classification models

The models based on PE-associated viral or bacterial markers were built using the randomForest (v4.7-1.1) package followed by five times of fivefold cross-validations, and their performances were evaluated based on the AUC that was calculated by the roc function (randomForest v4.7-1.1). The importance ordering of markers was obtained via the importance function (randomForest v4.7-1.1).

## Data Availability

The raw metagenomic sequencing data set for this study can be found in the European Nucleotide Archive (ENA) at EMBL-EBI under accession number PRJEB52189. All other data supporting the findings of the study are available in the paper and supplemental material.

## SUPPLEMENTAL MATERIAL

Figure S1 msphere.00676-23-s0001.tif Overview of the workflow for analyzing of gut bacteriome and virome.

Figure S2 msphere.00676-23-s0002.tif Classification of patients and controls based on individuals' parameters.

Legends msphere.00676-23-s0003.docx Supplemental material legends.

Supplemental tables msphere.00676-23-s0004.xlsx Tables S1-S8.

ASM does not own the copyrights to Supplemental Material that may be linked to, or accessed through, an article. The authors have granted ASM a non-exclusive, world-wide license to publish the Supplemental Material files. Please contact the corresponding author directly for reuse.
