# Supplementary figures and images for: Deep metagenomic characterization of the gut virome in pregnant women with preeclampsia
Source: mSphere. 2024 Mar 20;9(4):e00676-23. doi: 10.1128/msphere.00676-23 (PMC11036803; doi:10.1128/msphere.00676-23)

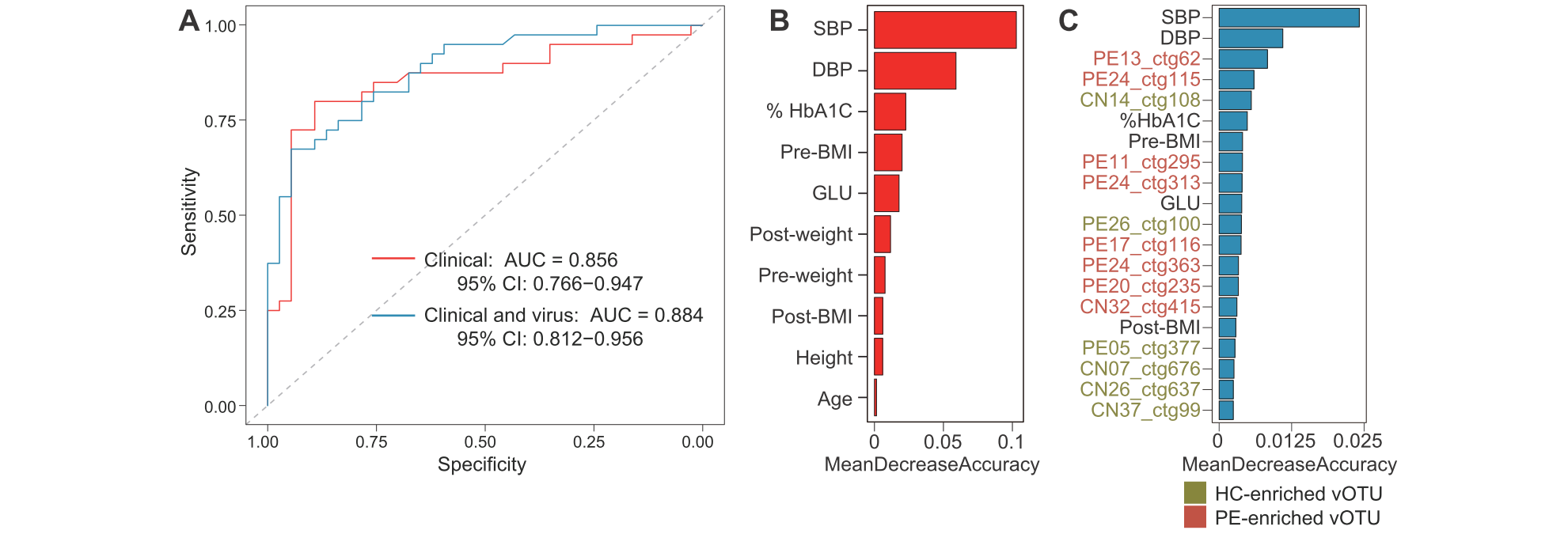

Supplement: Figure S2 — Classification of patients and controls based on individuals' parameters. [file msphere.00676-23-s0002.tif]
