# Supplementary material for: Deep metagenomic characterization of the gut virome in pregnant women with preeclampsia
Source: mSphere. 2024 Mar 20;9(4):e00676-23. doi: 10.1128/msphere.00676-23 (PMC11036803; doi:10.1128/msphere.00676-23)
Supplement: Legends — Supplemental material legends. [file msphere.00676-23-s0003.docx]

**Figure S1 Overview of the workflow for analyzing of gut bacteriome and virome.** PE, preeclampsia; QC, quality control; MAG, metagenome-assembled genome; SGB, species-level genome bin; ANI, average nucleotide identity; vOTU, viral operational taxonomic unit.

**Fgure S2 Classification of patients and controls based on individuals’ parameters. (A)** Random forest models for discriminating PE patients and healthy controls based on the individuals’ clinical parameters and the combination of gut viral signatures and clinical parameters. The area under the receiver-operating characteristic curve (AUC) and 95% confidence interval (CI) are shown. **(B-C)** Mean decrease in accuracy of the most important features in the clinical parameter model (B) and the combined model (C). For (C), bacteria that are enriched in patients and controls are labeled with red and green colors, respectively.

Table S1 Summary of the metagenomic assembly and viral identification of all samples used in this study.

Table S2 Detailed information of 8517 vOTUs.

Table S3 Richness and diversity of the gut virome of all fecal samples.

Table S4 Comparison of the gut virome of PE patients and healthy controls at the family level.

Table S5 Comparison of the gut virome of PE patients and healthy controls at the vOTU level.

Table S6 Detailed information of the KEGG-annotated genes of 27 PE-associated vOTUs.

Table S7 Detailed information of the PE network (left panel), control network (medium panel), and sharing network (right panel).

Table S8 Important scores of the gut viral and bacterial signatures in the viral model (left panel), bacterial model (medium panel), and virus-bacterium combinded model (right panel).
